# Deviant Dynamics of Resting State Electroencephalogram Microstate in Patients With Subjective Tinnitus

**DOI:** 10.3389/fnbeh.2018.00122

**Published:** 2018-06-22

**Authors:** Yuexin Cai, Dong Huang, Yanhong Chen, Haidi Yang, Chang-Dong Wang, Fei Zhao, Jiahao Liu, Yingfeng Sun, Guisheng Chen, Xiaoting Chen, Hao Xiong, Yiqing Zheng

**Affiliations:** ^1^Department of Otolaryngology, Sun Yat-sen Memorial Hospital, Sun Yat-sen University, Guangzhou, China; ^2^Institute of Hearing and Speech-Language Science, Sun Yat-sen University, Guangzhou, China; ^3^College of Mathematics and Informatics, South China Agricultural University, Guangzhou, China; ^4^School of Data and Computer Science, Sun Yat-sen University, Guangzhou, China; ^5^Department of Speech Language Therapy and Hearing Science, Cardiff Metropolitan University, Cardiff, United Kingdom; ^6^Department of Hearing and Speech Science, Xinhua College, Sun Yat-sen University, Guangzhou, China

**Keywords:** EEG, microstate, dynamics, tinnitus, mechanism

## Abstract

Given the importance of central reorganization and tinnitus, we undertook the current study to investigate changes to electroencephalogram (EEG) microstates and their association with the clinical symptoms in tinnitus. High-density (128 channel) EEG was used to explore changes in microstate features in 15 subjects with subjective tinnitus and 17 age-matched controls. Correlations between microstate parameters and subjective tinnitus symptoms were also analyzed. An increased presence of class A microstate and decreased presence of class D microstate were found in coverage and lifespan of microstate parameters in the tinnitus patients. Syntax analysis also demonstrated an aberrant pattern of activity, with reduced transitions from class D to class B in tinnitus patients. Moreover, a significant positive correlation of tinnitus loudness with increased lifespan of microstate class C was found. Significant differences in temporal characteristics and syntax of the EEG microstate classes were found at rest between tinnitus patients and the healthy subjects. Our study indicates that EEG microstates may provide a possible valuable method to study large-scale brain networks, which may in turn be beneficial to investigation of the neurophysiological mechanisms behind tinnitus.

## Introduction

Subjective tinnitus is defined as the perception of a phantom sound in the absence of a corresponding internal or external sound source (Jastreboff, 1990). It is a common clinical symptom experienced chronically by 10–15% of the adult population. Of these, approximately 1∼2% are affected severely (Langguth et al., 2013). At present, the main clinical interventions for tinnitus include counseling, cognitive behavioral therapy, tinnitus retraining therapy, and sound therapy. The therapeutic benefit of these interventions varies greatly among patients with subjective tinnitus (Langguth, 2015).

The effectiveness of tinnitus intervention requires a comprehensive understanding of the mechanisms of tinnitus. It is widely accepted that tinnitus is caused not only by peripheral hearing loss, but also as a result of aberrant neural activity in the central auditory pathway (Chen et al., 2014, 2015, 2016, 2017). Tinnitus patients with peripheral hearing loss show decreased signal output from the cochlea, which can result in tinnitus (Chen et al., 2016). Recent studies have indicated that even tinnitus patients with normal audiograms may have a “hidden hearing loss,” which may trigger tinnitus (Weisz et al., 2006). Functional imaging research shows hyperactivity in neurons along the auditory pathway in tinnitus patients (Arnold et al., 1996; Lanting et al., 2009). The activity of the central auditory system increases compensating for the reduction in cochlear output caused by deafferentation (Chen et al., 2017). These functional imaging studies also indicate that perception of tinnitus is related to aberrant activity in some non-auditory brain regions such as the prefrontal area and the limbic system which includes hippocampal and parahippocampal areas (Simonetti and Oiticica, 2015; Chen et al., 2016, 2017). Currently many pathophysiological models are offered to explain the generation and development of tinnitus, including central gain, thalamocortical dysrhythmia, neural synchrony, frontostriatal gating, noise-canceling deficit, global workspace, and precision coding (Llinas et al., 1999; Seki and Eggermont, 2003; Leaver et al., 2011; Schaette and McAlpine, 2011; De Ridder et al., 2014; Rauschecker et al., 2015; Sedley et al., 2016). The exact mechanism of subjective tinnitus is still the subject of significant debate. In this study, we explore alterations in central brain areas by tinnitus in a novel way to try to resolve the further central mechanisms behind tinnitus.

Electroencephalogram (EEG) is a technique that records electrical potentials within the brain using electrodes placed on the scalp. Resting-state EEG has been shown to be an inexpensive, non-invasive and high temporal resolution tool for studying tinnitus (Ingber and Nunez, 2011). Differences in EEG findings between tinnitus patients and those without tinnitus have been found (Moazami-Goudarzi et al., 2010). For example, EEG studies found an increase in gamma activity in the auditory cortex (Ortmann et al., 2011) whilst others revealed aberrant alterations in other brain regions outside the auditory cortex such as the parahippocampus and insula (Moazami-Goudarzi et al., 2010; Vanneste et al., 2010), indicating that tinnitus results from abnormal alterations in multiple brain regions.

There are several methods that can be used to extract information from EEG signals. One such mechanism – microstate analysis, identifies a series of quasi-stable microstates in the multichannel EEG signals. The microstate analysis simultaneously includes multichannel EEG recordings from across the scalp (Khanna et al., 2015). It can therefore partly compensate for the deficiency of EEG in identifying spatial resolution and analyze the abnormality of large-scale brain networks. Microstates can provide helpful information on global function to further understand brain neural activity at rest.

An increased number of studies have indicated that EEG microstates are significantly changed in some central neuropsychiatric diseases such as schizophrenia, dementia, depression, and panic disorder. Studies of EEG microstates in central neuropsychiatric diseases may provide a novel method for the detection of objective physiological biomarkers, to monitor the severity of disease, the evaluation of therapeutic effect and the design of targeted treatments (Khanna et al., 2015).

However, knowledge of microstate abnormalities in tinnitus is limited. A few EEG microstate studies have been conducted into auditory hallucination. For example, Kindler et al. (2011) found microstate D of the EEG to be significantly shorter in periods of hallucination when compared to non-hallucinating patients. As tinnitus and auditory hallucination share similar central cortex activity, with an increase in theta-gamma response during EEG measurement (Vanneste et al., 2013), and the generation of tinnitus involves several brain regions and multiple neural networks, the microstate analysis may be a powerful method to explore the neurophysiological mechanism of tinnitus over the whole brain. Therefore, the present study set out to explore alteration in EEG microstates in tinnitus patients with normal persons as controls. The specific purpose is to investigate the central mechanism of tinnitus and identify whether the EEG microstate is an effective method to study tinnitus. On the basis of central organization and alteration of cerebral connectivity after tinnitus, the hypothesis is proposed that significant changes of the resting state microstates would be found in tinnitus participants when compared to healthy subjects. Such differences could be used as an important electrophysiological marker of tinnitus.

## Materials and Methods

### Participants

Patients with tinnitus were recruited from the Ear, Nose and Throat clinic, Sun Yat-sen Memorial hospital, Sun Yat-sen University. Detailed selection criteria for inclusion and exclusion in this study are:

(1) They had sought clinical help for their tinnitus problem, which had lasted more than 3 months;
(2) They had no history of head trauma or central nervous system disorders;
(3) They had normal hearing thresholds or mild sensorineural hearing loss (the averaged hearing threshold ≤ 40 dB HL). All tinnitus patients with either current conductive hearing loss or previous middle ear surgery (e.g., mastoidectomy) as well as participants with moderate to severe hearing loss (i.e., the averaged hearing threshold ≥ 40 dB HL) were also excluded (Mohamad et al., 2016).
(4) Tinnitus patients with pulsatile tinnitus due to aberrant vascular malformation, Meniere’s disease, otosclerosis, chronic headache, neurological disorders such as brain tumors, traumatic brain injury or stroke and individual being treated for mental disorders as well as undergoing treatment (e.g., intake medication) for tinnitus during the study were not included in the study in order to increase the sample homogeneity.

The study group included 15 patients with tinnitus (11 male and 4 female; age Mean = 38.07 years, *SD* = 14.22 years) and 17 control subjects without tinnitus (10 male and 7 female; age Mean = 36.65 years, *SD* = 11.72 years). Prior to the experiment, a written consent form was signed by all participants after they had been properly informed of the experiment. The study was approved by the Institution Review Board of The Sun Yat-sen Memorial Hospital at Sun Yat-sen University of China.

### Routine Audiological Examinations

Routine audiological examination consisted of otoscopy, followed by pure-tone audiometry in which air conduction thresholds were measured for both ears at 125, 250, 500 Hz and 1.0, 2.0, 4.0 and 8.0 kHz, and bone conduction hearing thresholds were measured between 250 Hz and 4.0 kHz in a sound-proof booth. The mean hearing threshold is the average of hearing sensitivity at the frequencies of 500, 1000, 2000, and 4000 Hz. All participants had hearing thresholds measured using a pure tone audiometer (PTA). The audiological results showed average hearing thresholds, ranging from 250 to 8000 Hz at better than 20 dB HL for participants in control group.

### Tinnitus Specific Assessments

Patients were asked to describe their tinnitus characteristics, including duration and laterality (i.e., being in the right, left or both ears or central in the head). Tinnitus pitch and loudness matching measurements were performed ipsilaterally to the ear with predominant or louder tinnitus if there was a difference between the two sides. When the tinnitus was equally loud on both sides or was localized in the head, the matching tones were given to the ear with the better hearing. Otherwise, the ear was chosen randomly if there was no difference between the acuity of the two ears.

During the tinnitus pitch matching tests, the nine audiometric frequencies between 125 Hz and 8.0 kHz (i.e., 125, 250, 500 Hz and 1.0, 2.0, 3.0, 4.0, 6.0, and 8.0 kHz) were firstly used to roughly match the tinnitus pitch. Participants were initially asked to make a clear distinction between the perceptive tinnitus pitch and presented matching tones, and then they reported verbally whether the matching tone needed to go higher or lower until the exact matching tone or a close approximation to their tinnitus was obtained. The test tone was adjusted in a half-octave step. If there was no matching with a pure tone perceived by participants, narrow-band noise was used instead.

When the matching frequency was found, the level was initially set to 5 dB above the measured audiometric threshold to find an approximate tinnitus loudness level, then the level was adjusted in 1 dB step until the subject indicated that the tone matched the loudness of their tinnitus (Kim et al., 2016). The tinnitus loudness in decibels sensation level (dB SL) was computed by subtracting the presented sound intensity level in decibels hearing level (dB HL) with the auditory threshold at that frequency (De Ridder et al., 2015).

### Self-Reported Tinnitus Issues and Tinnitus Handicap Inventory (THI) Questionnaire

Tinnitus severity was assessed by the Tinnitus Handicap Inventory (THI). The THI questionnaire was provided to the patient prior to the experiment. The THI is a 25-item measure for evaluating the self-perceived level of handicap caused by tinnitus, based on a 0–100 increasing handicap scale (with 100 being total handicap and 0 being no handicap) (Newman et al., 1996).

### EEG Recordings

Recordings were made using a dense array EEG system with 128 channels and saved electronically with Electrical Geodesics, Inc. (EGI, Eugene), and a NetAmps 200 amplifier. The sampling rate was set to 1000 Hz and impedances were kept below 50 kΩ. The CZ electrode was used as reference for online recording. A resting EEG was obtained over approximately 7 min. During acquisition, the data was band pass filtered (0.1 Hz high-pass and 100 Hz low-pass). Subjects were instructed to sit on a chair in a comfortable position, to remain calm for the recording and to open their eyes and fixate a cross mark on the computer screen.

### Preprocessing of EEG Data

The raw data files from the EGI were transformed into mat file format ready for preprocessing with a EEGLAB for v13.0.0 toolbox by Matlab. Data were first re-referenced against the average reference by all the electrode and resampling rate to 500 Hz/s. EEG signals were amplified, band-pass-filtered to 0.5–80 Hz. A notch filter was implemented at 50 Hz to reduce the effect of the electric circuit on the EEG signal. Gross artifacts were manually removed from the raw EEGs after visual inspection, while components of muscle artifacts, eye movement, and heart beats were removed using an ICA-based correction process. After ICA correction, the signal was reconstructed and segmented into 2 s epochs. A minimum of 20 artifact-free epochs were randomly selected for microstate analysis.

### EEG Microstate Analysis

Microstate analysis followed the standard procedure used in earlier work (Koenig et al., 2002). Selected EEG epochs were digitally band pass filtered from 2 to 20 Hz. This bandpass filter was used as a consequence of previous studies (Koenig et al., 2002; Nishida et al., 2013), into the nature of microstates recorded in a multichannel array over the scalp and the alpha frequency band (8–12 Hz) of the multichannel resting-state EEG signal (Lehmann et al., 1987). Recently, most microstates studies are based on larger bandwidths with 2–20 Hz (Koenig et al., 2002; Kindler et al., 2011).

Due to the large number of original EEG maps, it is usual to reduce the EEG data to the time points at the local maxima of the global field power (GFP) before applying the clustering analysis process. The GFP reflects the potential variances across multi-electrodes at a specific time point. Let *K* denote the number of electrodes in the EEG data, *V_i_(t)* the potential of the *i*-th electrode at time *t*, the GFP can then be computed as:

$$\text{GFP}(t)=\sqrt{\frac{1}{K}\sum_{\text{i}=1}^{\text{K}}{\left(V_{\text{i}}(t)-V_{\text{mean}}(t)\right)}^{2}}$$

Where $V_{\text{mean}}(t)=\frac{1}{K}\sum_{\text{i}=1}^{\text{K}}V_{\text{i}}(t)$ is the mean of the instantaneous potentials across the electrodes. With the GFP computed, a set of topographic maps at the local minima of the GFP curve were extracted as the representative maps (König and Mellegarcia, 2009), upon which the clustering analysis was then performed to find the quasi-stable microstates in the EEG data. Clustering analysis is an important technique in the field of data mining and machine learning. Here, the purpose of clustering is to segment the scalp maps in the EEG data into several homogeneous subsets, each referred to as a microstate. It is suggested that the EEG maps can be typically clustered into four microstates, categorized as classes A, B, C, and D (König and Mellegarcia, 2009; Khanna et al., 2015). Specifically, we adopt a modified version of the classical *k*-means clustering algorithm to perform the segmentation. In terms of the categorization task, we use a top–down strategy to categorize the microstates of each subject and each group. First, the EEG maps (with maximum GFP) of all subjects from both the tinnitus group and the control group are collectively clustered into four grand subsets. With the mean map of each subset computed, the four grand subsets can be assigned to classes A, B, C, and D, respectively. Then, the EEG maps in the same group are clustered into four group subsets. Each of the group subsets is assigned to the same class as the grand subset that this group subset shows most similarity. Finally, the EEG maps of each subject (in the tinnitus group or the control group) can be clustered and assigned to one of the four microstate classes.

With the microstates of all subjects obtained, we proceeded to compute and analyze several parameters of the microstates, such as average lifespan, frequency of occurrence, coverage, topographical shape, amplitude, transition probabilities of microstates, etc. The average lifespan of a microstate is the average length of time when a microstate appears and remains stable. The frequency of occurrence of a microstate is the average number of times per second that a microstate occurs during a period of recording. The coverage of a microstate is the fraction of recording time that the microstate is dominant. There are four types of microstate topographical shape. The amplitude of a microstate is the mean GFP when microstate is dominant (Strelets et al., 2003; Nishida et al., 2013). The transition probability of a microstate means that the microstate is non-random and has the potential significance of sequence transfer (Lehmann et al., 2005).

### Statistical Analysis

To investigate the topographical differences of the microstate classes between the two groups we performed *t*-test on each class of microstates parameters. Pearson correlation analysis was performed to assess the association between the parameters of microstates and tinnitus subjective response. A *p* level of less than 0.05 (two-sided) was considered to be statistically significant. All statistical analyses were conducted using SPSS 13.0 software.

## Results

The demographic information of patients in the tinnitus group is shown in **Table 1**. There were no significant group differences between the tinnitus and control groups in terms of age (*t* = −0.310, df = 30, *p* = 0.759) and gender (*x*^2^ = 0.744, df = 1, *p* = 0.472). Tinnitus characteristic, including pitch, laterality, loudness, and hearing threshold are also given in **Table 1**.

**Table 1:** The landscape layout of participants with tinnitus.

Patient	Gender	Age (years)	Tinnitus laterality	Tinnitus duration (months)	Tinnitus pitch (Hz)	Tinnitus loudness	Hearing thresholds (dB HL) (L/R)	THI
ID1	Male	29	Right	36	8000	5	30/40	60
ID2	Male	28	Right	5	3000	22	6/16	54
ID3	Male	30	Left	6	4000	4	16/3	64
ID4	Female	59	Left	4	500	4	11/8	52
ID5	Male	36	Bilateral	6	8000	3	35/25	46
ID6	Female	47	Left	3	6000	15	26/13	78
ID7	Male	25	Left	12	8000	4	36/10	64
ID8	Female	60	Left	5	6000	16	40/6	50
ID9	Male	20	Bilateral	3	8000	10	8/8	24
ID10	Male	46	Bilateral	360	4000	0	24/24	82
ID11	Female	26	Left	3	2000	5	15/15	34
ID12	Male	34	Right	4	4000	0	18/17	50
ID13	Male	44	Left	3	4000	3	40/14	64
ID14	Male	63	Right	3	500	8	11/15	56
ID15	Male	24	Bilateral	4	4000	2	16/14	56

*L, left; R, right; THI, Tinnitus Handicap Inventory.*

**FIGURE 1:**
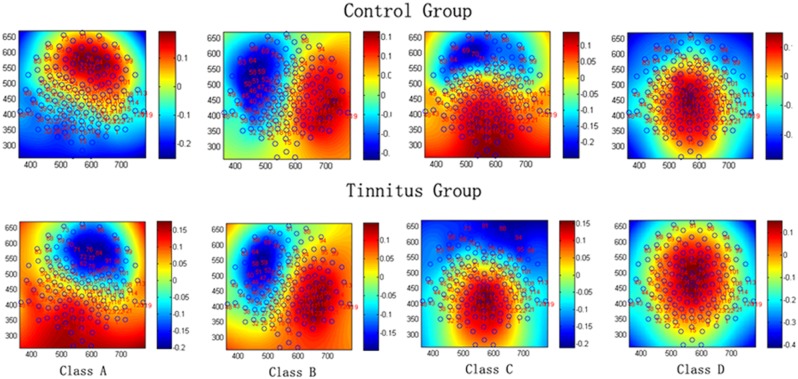
The spatial configuration of the four classes of microstates in the control group and tinnitus group.

The mean microstate maps obtained from both groups are shown in **Figure 1**. Similar to previous literature (Lehmann et al., 2005; Britz et al., 2010), four microstate topographies were found and classified as A, B, C, and D. Microstate class orientations were:

A - right-frontal left-posterior
B - left-frontal right-posterior
C - anterior–posterior
D - fronto-central extreme.

Independent sample *t*-tests between the tinnitus (*N* = 15) and control groups (*N* = 17) for each microstate parameter are detailed in **Table 2**. These parameters included the coverage, lifespan, amplitude and frequency of the microstates. The *t*-test revealed significant group differences in coverage and lifespan of classes A and D. With coverage, class A showed an increased presence (*t* = −2.748, df = 30, *p* = 0.01), whilst class D showed a decreased presence (*t* = 2.628, df = 30, *p* = 0.013) in the tinnitus group. There were no group differences in classes B and C. With lifespan, class A (*t* = −2.934, df = 30, *p* = 0.006) was significantly longer in the tinnitus patients than in controls, whilst the lifespan of class D (*t* = 2.244, df = 30, *p* = 0.032) in tinnitus patients was significantly shorter than in controls. There was no significant difference in class B or class C. No group differences in amplitude and frequency of the four microstates were found.

**Table 2:** Comparison of the microstate analysis results in patients with tinnitus and control subjects.

	Class A		Class B		Class C		Class D	
	Control (Mean ± SD)	Tinnitus (Mean ± SD)	Control (Mean ± SD)	Tinnitus (Mean ± SD)	Control (Mean ± SD)	Tinnitus (Mean ± SD)	Control (Mean ± SD)	Tinnitus (Mean ± SD)
Coverage (%)	0.14 ± 0.073	0.215 ± 0.081^∗∗^	0.22 ± 0.106	0.184 ± 0.088	0.309 ± 0.168	0.357 ± 0.15	0.327 ± 0.147	0.214 ± 0.084^∗^
Lifespan (ms)	328.84 ± 101.31	439.34 ± 111.76^∗∗^	438.4 ± 133.98	388.03 ± 116.15	600.29 ± 294.21	634.73 ± 281.14	559.69 ± 167.34	440.5 ± 127.19^∗^
Amplitude (uV)	0.206 ± 0.163	0.141 ± 0.146	0.152 ± 0.132	0.118 ± 0.204	0.176 ± 0.118	0.316 ± 0.688	0.147 ± 0.122	0.122 ± 0.177
Frequency	0.41 ± 0.167	0.575 ± 0.43	0.495 ± 0.163	0.544 ± 0.529	0.517 ± 0.182	0.572 ± 0.243	0.548 ± 0.186	0.572 ± 0.409

^∗^*and*^∗∗^*indicates the t-test was significant (p < 0.05, p < 0.001, respectively).*

**Table 3** demonstrates the correlations between changes in microstate features and tinnitus subjective symptoms. The only significant effect was found between the lifespan of class C and tinnitus loudness perception (*r* = 0.569, *p* = 0.027). No significant correlations were found in the changes of microstate features with THI in the tinnitus group (*p* > 0.05).

**Table 3:** Correlations between changes of microstates and tinnitus subjective symptoms.

	Microstate A *R*-value	Microstate B *R*-value	Microstate C *R*-value	Microstate D *R*-value
**Coverage (%)**
THI	0.062	−0.016	−0.090	0.263
Tinnitus loudness	−0.236	−0.464	0.286	−0.190
**Lifespan (ms)**				
THI	−0.099	−0.244	−0.287	−0.167
Tinnitus loudness	0.135	−0.157	0.569**^∗^**	0.279
**Amplitude (uV)**				
THI	−0.021	0.180	0.237	−0.063
Tinnitus loudness	0.001	0.129	−0.164	0.157
**Frequency**				
THI	0.182	0.125	0.056	0.202
Tinnitus loudness	−0.298	−0.292	0.015	−0.131

*THI, Tinnitus Handicap Inventory;*^∗^*Statistical significance.*

**FIGURE 2:**
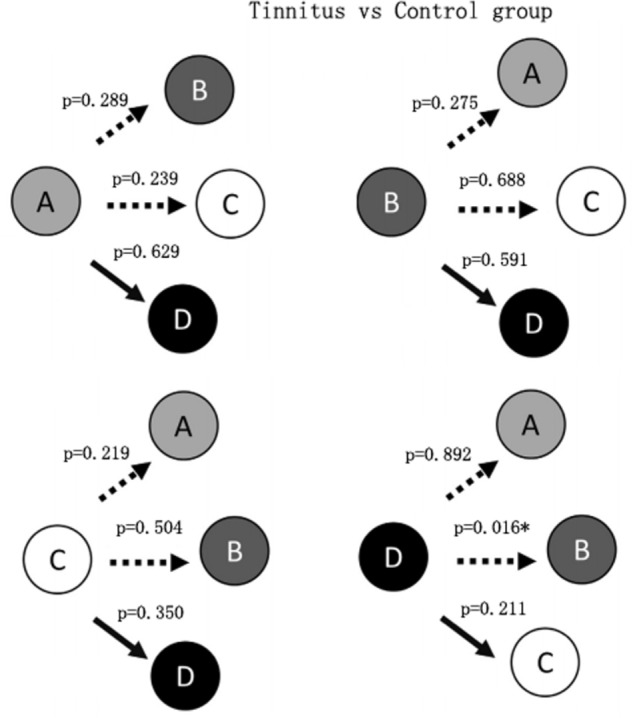
Schematic view of the syntax pattern. ^∗^Indicates significant difference (*p* < 0.05).

**Figure 2** shows that the probability of transition from class D microstate to class B microstate was significantly decreased (*p* = 0.016) in the tinnitus patients compared to controls. No other significant group differences in the probability of transition among microstate classes were found.

## Discussion

To our knowledge, this is the first study to investigate microstate dynamics in individuals with tinnitus. Significant differences were found in the temporal characteristics and syntax of the EEG microstate classes at rest for tinnitus patients when compared to healthy subjects. EEG microstate performance revealed a significantly increased presence of the class A microstate, and a decreased presence of the class D microstate. The syntax analysis also demonstrated an aberrant pattern of activity with reduced transitions from class D to class B in the patients. Moreover a significant positive correlation of tinnitus loudness with increased lifespan of microstate class C was found.

The time coverage and lifespan of microstate class A was significantly increased in our study. In the study by Britz et al. (2010), microstate class A was shown to correlate with activations in the bilateral superior and middle temporal gyrus areas that were related to phonological processing. Previous studies have shown hyperactivity in temporal areas and reorganization of the auditory cortex in tinnitus subjects (Diesch et al., 2010; Chen et al., 2015, 2016, 2017) and van der Loo et al. (2009) found enhancement of gamma oscillations over the auditory cortex that correlated with the tinnitus intensity. Given that tinnitus had a deficit on failing to stop attending to an essentially irrelevant signal (tinnitus sound), it may increases demands on auditory processing and attentional resources (Mohamad et al., 2016). Tinnitus may impoverish phonological input and interfere with the capability of phonological processing system to store auditory verbal information (Mohamad et al., 2016), which could add to the demand on the central executive with a resultant increase in the presence of microstate class A.

Reductions in time coverage and lifespan of microstate class D were also found in the tinnitus patients. Britz et al. (2010) suggested that microstate class D correlated with signals in the right-lateralized dorsal and ventral areas of the frontal and parietal cortex. These areas roughly corresponded to the central executive resting-state network. Neuroimaging evidence suggests that the frontal cortex, including the superior frontal gyrus and inferior frontal gyrus, exhibit increased neural activity in tinnitus patients, indicating that structural and functional differences in the frontal cortex may contribute to the mechanism and certain perceptual features of tinnitus (Burton et al., 2012). The inferior frontal gyrus serves as the core region of the central executive control network by regulating the attention allocation to tinnitus perception (Sridharan et al., 2008; Aron et al., 2014; Chen et al., 2017). The network involved in actively manipulating and maintaining attention, decision making and working-memory (Tomescu et al., 2014). Previous studies have reported impoverishment of attention execution and working memory in tinnitus. For example, Rossiter et al. (2006) suggested that tinnitus interferes with working memory and reduces cognitive capability to perform tasks that require effortful and attention. Heeren et al. (2014) also found that tinnitus subjects were unable to stop attending to the irrelevant tinnitus sound which might interfere with sustained executive attention. Therefore, the reduction of microstate class C indicates misengagement of the executive network which may be responsible for the self-reported tinnitus symptoms.

Another intriguing finding was the significant correlation between tinnitus loudness and the lifespan of microstate class C. This result was consistent with the finding by Tomescu et al. (2014), who showed a positive correlation between the presence of microstate class C and hallucinations in schizophrenia. Britz et al. (2010) stated that microstate class C was associated with a salience network, which was considered a large-scale neural network involving the detection and orientation of both internal and external stimuli. Recent studies assessing the functional and structural changes of tinnitus have found evidence of dysfunctional salience networks (Amaral and Langers, 2015). Network dysfunction would disrupt connectivity with the central executive attention network leading to deficits in cognitive processing, which could explain the positive tinnitus clinical symptoms (Tomescu et al., 2014). Therefore, the significant correlation shown in the present study indicates that the lifespan of microstate class C may be regarded as a possible objective and valuable biomarker to determine the severity of tinnitus.

Syntax analysis showed a significant decrease in the probability of transition from class D to class B microstates. Britz et al. (2010) suggested that the class B microstate was correlated with bilateral visual cortex areas. The decreased transition from the central executive network to a visual network was likely to result from greater demand on attentional resources to tinnitus sound which led to reduction of cognitive resources to visual attention. This result suggests that interventions that engage a visual attention network may provide relief from tinnitus-related systems due to the competition for attentional resources (Sedley et al., 2016).

Previous studies have attempted to discover the mechanisms behind tinnitus (Koenig et al., 2002; Langguth et al., 2013; Elgoyhen et al., 2015). Increased neuronal firing rate, enhanced neuronal synchrony, changes in tonotopic organization in central auditory pathways, and changes in non-auditory brain areas may be neuronal correlates of tinnitus (Langguth et al., 2013). Although several pathophysiological models have been proposed, none is able to explain all experimental and clinical findings. For example, one tinnitus model implicates the abnormal neuronal excitation of the auditory cortex due to cochlear damage (Schaette and Kempter, 2006). According to this model excitation in part of the auditory temporal cortex was increased to compensate for the auditory deafferentation and to maintain a stable neuronal activity (Elgoyhen et al., 2015). This model could be used to explain the results of the increased presence of the microstate A, which correlate with activation in bilateral superior and middle temporal cortex.

Another model to describe the development of tinnitus is that the central reorganization causes changes to structural and functional central network activity (Elgoyhen et al., 2015). The network dysfunction would disrupt connectivity with the central executive attention network leading to deficits in cognitive processing (Elgoyhen et al., 2015; Song et al., 2015). This model could therefore explain the significant correlation in the present study between the microstate class C and tinnitus loudness.

However, it is important to note that tinnitus is a highly heterogeneous condition with respect to hearing ability. Characteristics of the perceived sound are associated with variability of awareness and distress, duration and comorbidities (Langguth et al., 2013; Elgoyhen et al., 2015; Shore et al., 2016). Evidence shows that heterogeneity seem almost inevitable among tinnitus patients, even tinnitus patients with the same or similar hearing status conditions (e.g., tinnitus patients with normal hearing thresholds) (Zhao et al., 2014). As a result, it is extremely difficult to completely eliminate the heterogeneous factor, even when robust inclusion criteria are used. In addition, as the variability in the clinical presentation of the disorder is expected to be reflected by a similar development model, identifying the underlying neuronal mechanisms of tinnitus is challenging. The present study, due to its novelty and pilot study nature, will necessarily have limitations that require the results to be treated with caution. Small sample size is unable to rule out heterogeneous condition factors, such as hearing impairment. Future longitudinal research with larger sample sizes and comparisons of specific tinnitus subgroups (i.e., patients with tinnitus with different hearing status, distress levels, duration, laterality, and so on) is needed to develop a model of tinnitus, and examine the predictive value of EEG microstates for tinnitus mechanisms as well as its clinical relevance.

## Conclusion

Significant differences in temporal characteristics and syntax of the EEG microstate classes were found at rest between tinnitus patients and the healthy subjects. Our study indicates that EEG microstates may provide a possible valuable method to study large-scale brain networks, which may in turn be beneficial to investigation of the neurophysiological mechanisms behind tinnitus.

## Data Availability

No additional data are available. However, the original data that support the findings derived from this study can be requested by emailing yiqingzheng@hotmail.com.

## Ethics Statement

All participants were comprehensively informed about the background and the aim of the study. They all gave written informed consent in accordance with the Declaration of Helsinki. This study was carried out in accordance with the recommendations of guidelines of the ethics committee of Sun Yat-sen Memorial Hospital, Sun Yat-sen University. The protocol was approved by the ethics committee of Sun Yat-sen Memorial Hospital, Sun Yat-sen University.

## Author Contributions

YXC and DH contributed conception and design of the study. YHC, HY, and C-DW performed the experiments and collected the data. FZ and JL designed the plan of analysis. YS, GC, and XC performed the final analyses. YXC, DH and YZ drafted the manuscript and interpreted the results. All authors made substantive editorial contributions at all stages of manuscript preparation.

## Conflict of Interest Statement

The authors declare that the research was conducted in the absence of any commercial or financial relationships that could be construed as a potential conflict of interest.

## References

[B1] Amaral A. A. Langers D. R. (2015). Tinnitus-related abnormalities in visual and salience networks during a one-back task with distractors. *Hear. Res.* 326 15–29. 10.1016/j.heares.2015.03.006 25843940

[B2] Arnold W. Bartenstein P. Oestreiche E. Romer W. Schwaiger M. (1996). Focal metabolic activation in the predominant left auditory cortex in patients suffering from tinnitus: a PET study with [18F]deoxyglucose. *ORL J. Otorhinolaryngol. Relat. Spec.* 58 195–199. 10.1159/000276835 8883104

[B3] Aron A. R. Robbins T. W. Poldrack R. A. (2014). Inhibition and the right inferior frontal cortex: one decade on. *Trends Cogn. Sci.* 18 177–185. 10.1016/j.tics.2013.12.003 24440116

[B4] Britz J. Van De Ville D. Michel C. M. (2010). BOLD correlates of EEG topography reveal rapid resting-state network dynamics. *Neuroimage* 52 1162–1170. 10.1016/j.neuroimage.2010.02.052 20188188

[B5] Burton H. Wineland A. Bhattacharya M. Nicklaus J. Garcia K. S. Piccirillo J. F. (2012). Altered networks in bothersome tinnitus: a functional connectivity study. *BMC Neurosci.* 13:3. 10.1186/1471-2202-13-3 22217183 PMC3282646

[B6] Chen Y. C. Feng Y. Xu J. J. Mao C. N. Xia W. Ren J. (2016). Disrupted brain functional network architecture in chronic tinnitus patients. *Front. Aging Neurosci.* 8:174. 10.3389/fnagi.2016.00174 27458377 PMC4937025

[B7] Chen Y. C. Li X. Liu L. Wang J. Lu C. Q. Yang M. (2015). Tinnitus and hyperacusis involve hyperactivity and enhanced connectivity in auditory-limbic-arousal-cerebellar network. *eLife* 4:e06576. 10.7554/eLife.06576 25962854 PMC4426664

[B8] Chen Y. C. Wang F. Wang J. Bo F. Xia W. Gu J. P. (2017). Resting-state brain abnormalities in chronic subjective tinnitus: a meta-analysis. *Front. Hum. Neurosci.* 11:22. 10.3389/fnhum.2017.00022 28174532 PMC5258692

[B9] Chen Y. C. Zhang J. Li X. W. Xia W. Feng X. Gao B. (2014). Aberrant spontaneous brain activity in chronic tinnitus patients revealed by resting-state functional MRI. *Neuroimage Clin.* 6 222–228. 10.1016/j.nicl.2014.09.011 25379434 PMC4215464

[B10] De Ridder D. Congedo M. Vanneste S. (2015). The neural correlates of subjectively perceived and passively matched loudness perception in auditory phantom perception. *Brain Behav.* 5:e331. 10.1002/brb3.331 25874164 PMC4389054

[B11] De Ridder D. Vanneste S. Weisz N. Londero A. Schlee W. Elgoyhen A. B. (2014). An integrative model of auditory phantom perception: tinnitus as a unified percept of interacting separable subnetworks. *Neurosci. Biobehav. Rev.* 44 16–32. 10.1016/j.neubiorev.2013.03.021 23597755

[B12] Diesch E. Andermann M. Flor H. Rupp A. (2010). Functional and structural aspects of tinnitus-related enhancement and suppression of auditory cortex activity. *Neuroimage* 50 1545–1559. 10.1016/j.neuroimage.2010.01.067 20114077

[B13] Elgoyhen A. B. Langguth B. De Ridder D. Vanneste S. (2015). Tinnitus: perspectives from human neuroimaging. *Nat. Rev. Neurosci.* 16 632–642. 10.1038/nrn4003 26373470

[B14] Heeren A. Maurage P. Perrot H. De Volder A. Renier L. Araneda R. (2014). Tinnitus specifically alters the top-down executive control sub-component of attention: evidence from the Attention Network Task. *Behav. Brain Res.* 269 147–154. 10.1016/j.bbr.2014.04.043 24793493

[B15] Ingber L. Nunez P. L. (2011). Neocortical dynamics at multiple scales: EEG standing waves, statistical mechanics, and physical analogs. *Math. Biosci.* 229 160–173. 10.1016/j.mbs.2010.12.003 21167841

[B16] Jastreboff P. J. (1990). Phantom auditory perception (tinnitus): mechanisms of generation and perception. *Neurosci. Res.* 8 221–254. 10.1016/0168-0102(90)90031-9 2175858

[B17] Khanna A. Pascual-Leone A. Michel C. M. Farzan F. (2015). Microstates in resting-state EEG: current status and future directions. *Neurosci. Biobehav. Rev.* 49 105–113. 10.1016/j.neubiorev.2014.12.010 25526823 PMC4305485

[B18] Kim T. S. Yoo M. H. Lee H. S. Yang C. J. Ahn J. H. Chung J. W. (2016). Short-term changes in tinnitus pitch related to audiometric shape in sudden sensorineural hearing loss. *Auris Nasus Larynx* 43 281–286. 10.1016/j.anl.2015.10.001 26620396

[B19] Kindler J. Hubl D. Strik W. K. Dierks T. Koenig T. (2011). Resting-state EEG in schizophrenia: auditory verbal hallucinations are related to shortening of specific microstates. *Clin. Neurophysiol.* 122 1179–1182. 10.1016/j.clinph.2010.10.042 21123110

[B20] Koenig T. Prichep L. Lehmann D. Sosa P. V. Braeker E. Kleinlogel H. (2002). Millisecond by millisecond, year by year: normative EEG microstates and developmental stages. *Neuroimage* 16 41–48. 10.1006/nimg.2002.1070 11969316

[B21] König T. Mellegarcia L. (2009). “Statistical analysis of multichannel scalp field data,” in *Proceedings of the EGU General Assembly Conference* Vienna 10.1017/CBO9780511596889.009

[B22] Langguth B. (2015). Treatment of tinnitus. *Curr. Opin. Otolaryngol. Head Neck Surg.* 23 361–368. 10.1097/MOO.0000000000000185 26261868

[B23] Langguth B. Kreuzer P. M. Kleinjung T. De Ridder D. (2013). Tinnitus: causes and clinical management. *Lancet Neurol.* 12 920–930. 10.1016/S1474-4422(13)70160-1 23948178

[B24] Lanting C. P. de Kleine E. van Dijk P. (2009). Neural activity underlying tinnitus generation: results from PET and fMRI. *Hear. Res.* 255 1–13. 10.1016/j.heares.2009.06.009 19545617

[B25] Leaver A. M. Renier L. Chevillet M. A. Morgan S. Kim H. J. Rauschecker J. P. (2011). Dysregulation of limbic and auditory networks in tinnitus. *Neuron* 69 33–43. 10.1016/j.neuron.2010.12.002 21220097 PMC3092532

[B26] Lehmann D. Faber P. L. Galderisi S. Herrmann W. M. Kinoshita T. Koukkou M. (2005). EEG microstate duration and syntax in acute, medication-naive, first-episode schizophrenia: a multi-center study. *Psychiatry Res.* 138 141–156. 10.1016/j.pscychresns.2004.05.007 15766637

[B27] Lehmann D. Ozaki H. Pal I. (1987). EEG alpha map series: brain microstates by space-oriented adaptive segmentation. *Electroencephalogr. Clin. Neurophysiol.* 67 271–288. 10.1016/0013-4694(87)90025-3 2441961

[B28] Llinas R. R. Ribary U. Jeanmonod D. Kronberg E. Mitra P. P. (1999). Thalamocortical dysrhythmia: a neurological and neuropsychiatric syndrome characterized by magnetoencephalography. *Proc. Natl. Acad. Sci. U.S.A.* 96 15222–15227. 10.1073/pnas.96.26.15222 10611366 PMC24801

[B29] Moazami-Goudarzi M. Michels L. Weisz N. Jeanmonod D. (2010). Temporo-insular enhancement of EEG low and high frequencies in patients with chronic tinnitus. QEEG study of chronic tinnitus patients. *BMC Neurosci.* 11:40. 10.1186/1471-2202-11-40 20334674 PMC2858736

[B30] Mohamad N. Hoare D. J. Hall D. A. (2016). The consequences of tinnitus and tinnitus severity on cognition: a review of the behavioural evidence. *Hear. Res.* 332 199–209. 10.1016/j.heares.2015.10.001 26523370

[B31] Newman C. W. Jacobson G. P. Spitzer J. B. (1996). Development of the tinnitus handicap inventory. *Arch. Otolaryngol. Head Neck Surg.* 122 143–148. 10.1001/archotol.1996.01890140029007 8630207

[B32] Nishida K. Morishima Y. Yoshimura M. Isotani T. Irisawa S. Jann K. (2013). EEG microstates associated with salience and frontoparietal networks in frontotemporal dementia, schizophrenia and Alzheimer’s disease. *Clin. Neurophysiol.* 124 1106–1114. 10.1016/j.clinph.2013.01.005 23403263

[B33] Ortmann M. Muller N. Schlee W. Weisz N. (2011). Rapid increases of gamma power in the auditory cortex following noise trauma in humans. *Eur. J. Neurosci.* 33 568–575. 10.1111/j.1460-9568.2010.07542.x 21198988

[B34] Rauschecker J. P. May E. S. Maudoux A. Ploner M. (2015). Frontostriatal gating of tinnitus and chronic pain. *Trends Cogn. Sci.* 19 567–578. 10.1016/j.tics.2015.08.002 26412095 PMC4587397

[B35] Rossiter S. Stevens C. Walker G. (2006). Tinnitus and its effect on working memory and attention. *J. Speech Lang. Hear. Res.* 49 150–160. 10.1044/1092-4388(2006/012) 16533080

[B36] Schaette R. Kempter R. (2006). Development of tinnitus-related neuronal hyperactivity through homeostatic plasticity after hearing loss: a computational model. *Eur. J. Neurosci.* 23 3124–3138. 10.1111/j.1460-9568.2006.04774.x 16820003

[B37] Schaette R. McAlpine D. (2011). Tinnitus with a normal audiogram: physiological evidence for hidden hearing loss and computational model. *J. Neurosci.* 31 13452–13457. 10.1523/JNEUROSCI.2156-11.2011 21940438 PMC6623281

[B38] Sedley W. Friston K. J. Gande P. E. Kumar S. Griffiths T. D. (2016). An integrative tinnitus model based on sensory precision. *Trends Neurosci.* 39 799–812. 10.1016/j.tins.2016.10.004 27871729 PMC5152595

[B39] Seki S. Eggermont J. J. (2003). Changes in spontaneous firing rate and neural synchrony in cat primary auditory cortex after localized tone-induced hearing loss. *Hear. Res.* 180 28–38. 10.1016/S0378-5955(03)00074-1 12782350

[B40] Shore S. E. Roberts L. E. Langguth B. (2016). Maladaptive plasticity in tinnitus–triggers, mechanisms and treatment. *Nat. Rev. Neurol.* 12 150–160. 10.1038/nrneurol.2016.12 26868680 PMC4895692

[B41] Simonetti P. Oiticica J. (2015). Tinnitus neural mechanisms and structural changes in the brain: the contribution of neuroimaging research. *Int. Arch. Otorhinolaryngol.* 19 259–265. 10.1055/s-0035-1548671 26157502 PMC4490922

[B42] Song J. J. Vanneste S. Schlee W. Van de Heyning P. De Ridder D. (2015). Onset-related differences in neural substrates of tinnitus-related distress: the anterior cingulate cortex in late-onset tinnitus, and the frontal cortex in early-onset tinnitus. *Brain Struct. Funct.* 220 571–584. 10.1007/s00429-013-0648-x 24135769

[B43] Sridharan D. Levitin D. J. Menon V. (2008). A critical role for the right fronto-insular cortex in switching between central-executive and default-mode networks. *Proc. Natl. Acad. Sci. U.S.A.* 105 12569–12574. 10.1073/pnas.0800005105 18723676 PMC2527952

[B44] Strelets V. Faber P. L. Golikova J. Novototsky-Vlasov V. Koenig T. Gianotti L. R. (2003). Chronic schizophrenics with positive symptomatology have shortened EEG microstate durations. *Clin. Neurophysiol.* 114 2043–2051. 10.1016/S1388-2457(03)00211-6 14580602

[B45] Tomescu M. I. Rihs T. A. Becker R. Britz J. Custo A. Grouiller M. (2014). Deviant dynamics of EEG resting state pattern in 22q11.2 deletion syndrome adolescents: a vulnerability marker of schizophrenia? *Schizophr. Res.* 157 175–181. 10.1016/j.schres.2014.05.036 24962438

[B46] van der Loo E. Gais S. Congedo M. Vanneste S. Plazier M. Menovsky T. (2009). Tinnitus intensity dependent gamma oscillations of the contralateral auditory cortex. *PLoS One* 4:e7396. 10.1371/journal.pone.0007396 19816597 PMC2754613

[B47] Vanneste S. Plazier M. van der Loo E. Van de Heyning P. Congedo M. De Ridder D. (2010). The neural correlates of tinnitus-related distress. *Neuroimage* 52 470–480. 10.1016/j.neuroimage.2010.04.029 20417285

[B48] Vanneste S. Song J. J. De Ridder D. (2013). Tinnitus and musical hallucinosis: the same but more. *Neuroimage* 82 373–383. 10.1016/j.neuroimage.2013.05.107 23732881

[B49] Weisz N. Hartmann T. Dohrmann K. Schlee W. Norena A. (2006). High-frequency tinnitus without hearing loss does not mean absence of deafferentation. *Hear. Res.* 222 108–114. 10.1016/j.heares.2006.09.003 17079102

[B50] Zhao F. Stephens S. D. Ishak W. S. Meyer-Bisch C. (2014). The characteristics of Audioscan and DPOAE measures in tinnitus patients with normal hearing thresholds. *Int. J. Audiol.* 53 309–317. 10.3109/14992027.2013.868047 24495275

